# Soil Bacteria Isolated From Tunisian Arid Areas Show Promising Antimicrobial Activities Against Gram-Negatives

**DOI:** 10.3389/fmicb.2018.02742

**Published:** 2018-11-13

**Authors:** Zina Nasfi, Henrik Busch, Stefan Kehraus, Luis Linares-Otoya, Gabriele M. König, Till F. Schäberle, Rafik Bachoual

**Affiliations:** ^1^Laboratory of Plant Improvement and Valorization of Agroresources, National School of Engineering of Sfax, Sfax, Tunisia; ^2^Institute for Insect Biotechnology, Justus-Liebig-University Giessen, Giessen, Germany; ^3^Faculty of Sciences of Gabès, University of Gabès, Gabès, Tunisia; ^4^Institute for Pharmaceutical Biology, University of Bonn, Bonn, Germany; ^5^Department of Bioresources of the Fraunhofer Institute for Molecular Biology and Applied Ecology, Giessen, Germany

**Keywords:** *Bacilli*, natural products, antibiotics, carboline, fungicides

## Abstract

Arid regions show relatively fewer species in comparison to better-watered biomes, but the competition for the few nutrients is very distinct. Here, in total 373 bacterial strains were isolated from rhizospheric soils obtained from three different sampling sites in Tunisia. Their potential for the production of antimicrobial compounds was evaluated. Bacterial strains, showing antibacterial activity against pathogenic bacteria, were isolated from all three sites, one strain from the Bou-Hedma national park, 15 strains from Chott-Djerid, and 13 strains from Matmata, respectively. The dominant genus was *Bacillus*, with 27 out of 29 strains. Most interestingly, 93% of the isolates showed activity against Gram-positive and Gram-negative test bacteria. Strain *Bacillus* sp. M21, harboring high inhibitory potential, even against clinical isolates of Gram-negative bacteria, was analyzed in detail to enable purification and identification of the bioactive compound responsible for its bioactivity. Subsequent HPLC-MS and NMR analyses resulted in the identification of 1-acetyl-β-carboline as active component. Furthermore, fungicides of the bacillomycin and fengycin group, which in addition show antibiotic effects, were identified. This work highlights the high potential of the arid-adapted strains for the biosynthesis of specialized metabolites and suggest further investigation of extreme environments, since they constitute a promising bioresource of biologically active compounds.

## Introduction

The discovery of antibiotics to treat infectious diseases has revolutionized the field of medicine in the mid-twentieth century (Wohlleben et al., 2016). However, due to the misuse of antibiotics or extensive use for clinical and veterinary purposes, many of the relevant pathogens became resistant to antibiotic therapy (Ventola, 2015). These pathogens have accumulated a large number of resistance elements by mutational adaptations, acquisition of genetic material, or alteration of gene expression. This resulted in (i) modification of the antimicrobial target, (ii) a decrease in drug-uptake, (iii) activation/increase of efflux mechanisms to extrude the harmful molecule, or (iv) global changes in important metabolic pathways via modulation of regulatory networks (Munita and Arias, 2016).

The genes encoding for the respective resistance mechanism can be found both, within the genome and on plasmids; thereby, greatly limiting the therapeutic options (Wright, 2012). The number of resistant bacterial isolates increases at an alarming rate (Frieri et al., 2017), threatening treatment options of modern medicine. Hence, there is an immense need for the discovery and development of novel antibiotics with resistance-breaking properties to effectively target the multi drug resistant (MDR) pathogen bacteria that cause life-threatening infections (Morens and Fauci, 2013).

Medicinal drugs based on natural products obtained from microorganisms are playing the most important role in the treatment of bacterial infections and appear to be still the most promising source of future antibiotics (Kumar and Kumar, 2016). This became clear, since the standard research approach performed in the recent decades, from gene via target using high-throughput screening to generate a lead, delivered no innovative antibiotics. Chemical compound libraries, optimized for oral bioavailability, have non-suitable physicochemical properties, especially for passing through Gram-negative cell membranes. On the other hand, natural products, optimized for bacterial activity, but not for human application can be associated with toxicity and low *in vivo* activity. A drawback in natural product research is that it seems like the low-hanging fruits are already harvested. Often, known compounds had been re-isolated. Combined with the fact that the economic value of a new antibiotic can be close to zero, thereby facing development costs of around 1.000 Mio€, since for innovative novel resistance breaking antibiotics, only small margins can be expected. An innovative novel antibiotic will receive the status of a reserve antibiotic, which will result in relatively low sales figures. Therefore, companies and research groups left the field: Today only 50 groups worldwide are active in antibiotic research with a total of ∼500 people (The Boston Consulting Group, 2017).

To increase the chance of success for bioprospecting projects, which aim to identify novel lead structures for antibiotic development, these must be based on a good rational. In nature, there will be still many different potential sources to discover such leads. Rhizospheric soil in general, with its enormous biological diversity, remains a most important target for screening projects; since there is a universal dissemination of antibiotics among rhizospheric microorganisms. The latter seem to shape the microbiome of the specific biological niche and using specialized metabolites of interest for communication and antagonism (Raaijmakers and Mazzola, 2012; Ghanmi et al., 2016). Many pharmaceutically important antibiotics have been identified in the past from this bioresource, e.g., vancomycin produced by *Streptomyces orientalis* isolated from a soil sample from Borneo (Griffith, 1981), kanamycin produced by a soil bacterium *Streptomyces kanamyceticus* (Umezawa et al., 1957), and erythromycin first isolated in 1952 from the soil bacterium *Saccharopolyspora erythraea* (Staunton and Wilkinson, 1997). Bacterial genera reported so far as a bioresource with a high chance to detect compounds of interest are *Actinomycetes* (Hotam et al., 2013; Tiwari and Gupta, 2013), *Bacilli* (Sumi et al., 2015), and *Pseudomonads* (Mukherjee et al., 2014; de Oliveira et al., 2016). In the present project so far unexplored arid sampling sites of Southern Tunisia were investigated, since the arid environment results in high competition between organisms. Several strains with antimicrobial properties were isolated and characterized. From one isolated *Bacillus* strain, an antimicrobial compound was isolated and further bioactive natural products were identified by LC/MS.

## Materials and Methods

### Sampling Sites

**FIGURE 1:**
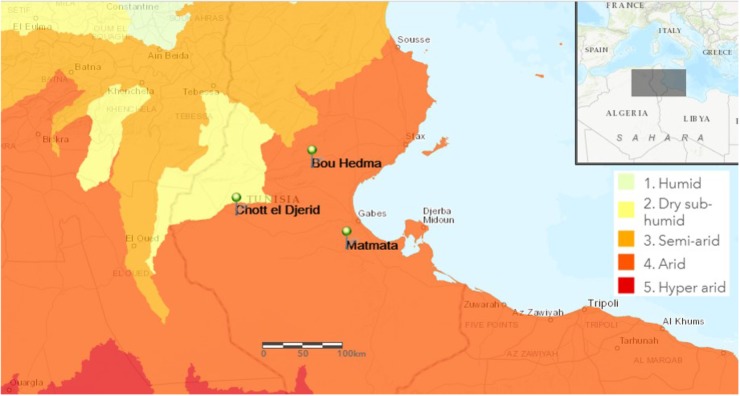
Map of Tunisia showing the location of the sampling sites. Bou-Hedma national park (Sidibouzid), Chott-Djerid (Tozeur), and Matmata (Gabès).

Samples were collected from different arid areas located in South Tunisia (Figure 1). Three rhizospheric soil samples, of herbaceous vegetation, were collected aseptically from Matmata (33°54′86^′′^ N, 9°96′13^′′^ E), the national park of Bou-Hedma (34°47′45^′′^ N, 9°48′21^′′^ E) and an arid shallow aquifer in Chott-Djerid (33°94′16^′′^ N, 8°44′52^′′^ E). Matmata has an arid climate with hot, dry summers and a short, highly variable humid period in winter with mean annual precipitation < 150 mm (Dearing et al., 1996). Bou-Hedma national park has a low arid climate with an approximate mean annual rainfall of 180 mm, a mean annual temperature of 17.2°C, and minimum and maximum monthly mean temperatures of 3.8°C in December and 36.2°C in July, respectively (Le Houérou, 2001). The Chott-Djerid is a flat area, with a mean altitude of 15 m. The mean annual rainfall for the area is around 100 mm (Richards and Vita-Finzi, 1982). The texture of the three soil samples was sandy to sandy-loamy.

### Soil Sampling and Isolation of Bacterial Strains

Each soil collection was made from 10 to 15 cm depth below ground surface. Samples were placed in sterile flasks and transported immediately to the laboratory, where they were air-dried for 2 h at 45°C and sieved prior use. For isolation purpose 1 g of the dried samples was dissolved in 10 mL of sterile distilled water. Soil samples were shaken vigorously for 2 min and the suspensions were treated by heat for 10 min at 80°C, except the soil suspension of Chott-Djerid. A serial dilution in sterile physiological salt solution (0.9% NaCl) up to 10^−4^ was prepared and an aliquot of 0.1 mL was spread over Luria-Bertani (LB) medium agar plates. Plates were incubated at 37°C for 24 h. After incubation, colonies were isolated, recorded and subjected to antimicrobial activity screening. Purified strains that inhibited the growth of at least one of the test microorganisms, were selected, cryo-conserved in 20% v/v glycerol stocks, and stored at −20°C. The code used for the isolates is (B) for Bou-Hedma national park, (C) for Chott-Djerid, and (M) for Matmata.

### Growth Conditions

The isolated bacterial strains were cultivated in LB broth, which contains 10 g peptone, 5 g yeast extract and 10 g NaCl per L. The pH was adjusted to 7 with 0.01M HCl and 0.01M NaOH. Incubation was performed at 37°C and agitation of 200 rpm. The cultures were inoculated into broth medium with 1% (v/v) inoculum.

### Bacterial Strains

Gram-positive test bacteria used: The reference strain *Staphylococcus aureus* ATCC 29213, and three clinical isolates, i.e., *S. aureus*, *S. epidermidis*, and *S. saprophyticus*. The last three bacterial strains were kindly provided by the Tunisian Sahloul hospital at Sousse. Gram-negative test bacteria: Two clinical isolates of *Salmonella typhimurium* and six *Escherichia coli* strains (one clinical isolate *E. coli* MA, three reference strains *E. coli* ATCC 25922, ATCC 35218 and KL 16; two *in vitro* mutants *E. coli* KL 16.2a and *E. coli* KL 16.2b, derived from the reference strain *E. coli* KL 16 by selection at twofold MIC of ciprofloxacin. These strains carry point mutations in the *gyrA* and *parC* genes (Ser83Leu in GyrAand Ser80Ile in ParC, respectively). *E. coli* MA, carry three mutations, two in *gyrA* (Ser83Leu and Asp87Asn) and one in *parC* (Ser80Ile). The mutant strains were recovered from patients with urinary tract infection (Bachoual et al., 1998); and a clinical *E. coli* isolate from the strain collection of the Tunisian Sahloul hospital at Sousse.

### Antimicrobial Activity

#### Primary Screening

The antibacterial activity of pure isolates was determined by spot assay on Mueller Hinton (MH) agar. In brief, the test bacteria, i.e., *E. coli* ATCC 25922 and *S. aureus* ATCC 29213 were plated on agar plates before isolated soil bacteria were spotted on top. Plates were incubated for 24 h at 37°C. After incubation, bacterial spots showing an inhibition zone were selected for a secondary screening, which was performed in triplicate by disk agar diffusion on MH agar plates against several test organisms. Cell free supernatant (50 μL) was used to saturate a sterilized Whatman filter paper disc (6 mm), allowed to dry at room temperature and placed onto agar plates inoculated with 10^7^ CFU/mL of the test bacteria. Plates were incubated at 37°C for 24 h. Antimicrobial activity was evaluated by measuring the diameter of the inhibition zone around each paper disc. All tests were performed in triplicate. The average results are presented in Supplementary Table 1.

#### Screening of Large-Scale Extract

Strain *Bacillus* M21a was fermented as described at point 2.9. The organic phase of this fermentation was obtained by liquid/liquid extraction between fermentation broth and ethyl acetate. The resulting organic phase was subjected to FLASH chromatography (BUCHI Reveleris X2, column silica 40 g). The collected fractions were tested for their antibacterial activity on agar plates and on 96 well plates. For the 96 well plate assay, wells were loaded with respective amounts of 200, 100, and 50 μg of extract. The antibiotic ampicillin (final concentration of 20, 10, and 5 μg per well) and DMSO (10, 5, and 2.5 μL per well) were used as positive and negative control against the test bacteria, respectively. Volume per well was 200 μL. After loading the samples, the 96 well plate was measured at 600 nm to determine the absorbance (time T0). At T0, the OD_600_ was 0.1. After incubation at 30°C for 24 h, the absorbance was measured again (time T1). The growth inhibition of the test bacteria (in %) was calculated by using the formula: % inhibition = (OD_T1_ − OD_T0_)/[AV (− control)] ^∗^ 100. Wells with values ≤ 30% were considered to have an antibacterial effect.

### Identification of the Producer Strains

The isolates were preliminary characterized based on colony morphology and cell characteristics. Subsequently, identification by 16S rDNA sequencing was performed. Therefore, the 16S rDNA of the isolates was amplified by PCR using universal primers pA (5′AGAGTTTGATCCTGGCTCAG 3′) and pH (5′AAGGAGGTGATCCAGCCCCA 3’). Amplification was done according to the following profile: an initial denaturation step at 94°C for 10 min, followed by 30 amplification cycles of 94°C for 45 s, 56°C for 45 s, and 72°C for 2 min, and a final extension step of 72°C for 4 min. The PCR product was subjected to agarose gel electrophoresis at 80V for 30 min. The DNA fragment in the agar was visualized by ultraviolet fluorescence after ethidium bromide staining, then excised and purified using a kit (Wizard^®^ SV Gel and PCR Clean Up System, Promega).

The resulting fragments were cloned into pGEM-T vector (Promega, Madison, WI, United States), introduced into chemical competent *E. coli* XL-1 Blue cells by transformation. The plasmids were isolated using the PureYieldMiniprep kit (Promega, Madison, WI, United States) and the inserts were sequenced from both sides using T7and SP6 primers (GATC BioTech AG Company, Konstanz, Germany). Vector fragments in the sequencing result were removed using VecScreen-Blast and the full 16S rDNA were assembled. The 16S rDNA sequences were blasted with the available 16S rRNA gene sequences contained in the GenBank database^1^.

### Gram Staining and Mobility Test of Bioactive Bacterial Isolates

A smear of bacterial isolate was applied onto a clean glass slide and heated gently over a flame. The fixed bacteria were covered with a thin film of crystal violet for 1 min and washed gently under slow running water. Gram’s iodine solution was flooded over the glass slide for 1 min and washed with water. Ethanol (80%) was used to decolorize the sample. Counter staining was performed with fuchsine for 2 min. Glass slides were washed, air dried, and analyzed.

For the bacterial mobility test, a small drop of bacterial culture was placed in the center of a clean microscope slide, covered with a cover glass, and then examined microscopically for the motility of the bacterial cells.

### Phylogenetic Analysis

Sequences of each isolate were refined using BioEdit sequence Alignment Editor (Hall, 1999), in which the sequences obtained from both primers were assembled to obtain consensus sequences. To analyze the relationships of the isolates to known bacterial species, the 29 sequences from this study and sequences of type strains, which had the closest relationship, were initially aligned using the MUSCLE Multiple alignment (Edgar, 2004). Based on the homology of 16S rDNA sequences, the evolutionary distances were computed through MEGA (version 7.0) program (Kumar et al., 2016) with p-distance using neighbor-joining method (Saitou and Nei, 1987). Gaps were treated as missing data. Further, the bootstrap values were calculated from 1000 replications to represent the evolutionary history of the bacterial isolates (Felsenstein, 1985).

### Isolation of 1-acetyl-β-carboline

Two *Bacillus* strains, i.e., M21 (Nasfi et al., 2018) and M21a, were identified positive for the production of 1-acetyl-β-carboline. For isolation, both strains were inoculated into LB broth and incubated at 30°C under shaking condition. However, strain M21 lost its viability and therefore all downstream steps were performed using strain M21a. In order to isolate the bioactive compound(s), a single colony of *Bacillus* sp. M21a was subcultured in LB broth and incubated at 30°C for 24 h. The resulting culture was used to inoculate forty 5 L flasks containing 1 L LB broth each. Flasks were incubated at 30°C for 48 h with agitation at 140 rpm in presence of 20 g/L autoclaved amberlite XAD-16, a polymeric resin often used as a first step in downstream processing to separate bioactive compounds from bacterial growth media. The resin was collected by filtration and washed sequentially with 5 L distilled water to remove impurities. Thereafter, the resin was resuspended in a mixture of MeOH-acetone and maintained at 26°C for 18 h with agitation followed by filtration. The resulting filtrate was concentrated by a rotary evaporator at 40°C under vacuum to yield the crude extract. This extract was partitioned using liquid-liquid separation between MeOH 60% in water and DCM. The organic phase was bioassayed for activity by disc diffusion method against *E. coli* and *B. megaterium* and was subsequently subjected to successive chromatographic purification. Medium pressure (“Flash”) chromatography was performed on a silica gel 40 column, using an elution gradient with A: Petroleum ether and B: acetone with A:B from 100–0 to 0–100% (Reveleris Silica 40 μm, 12 g; Flow rate 40 mL/min; 400 mg – 8 g sample). Purity of the active fraction(s) was further examined using HPLC with an RP column (Xterra RP 18–5 μm). At a concentration of 5 mg/mL, a 20 μL aliquot was applied onto the RP column. The mobile phase consisted of an isocratic gradient of 60% MeOH (solvent B) in Milli-Q water (solvent A). The flow rate of the mobile phase was set at 0.8 mL/min for 45 min. Elution was monitored at a wavelength of 210 nm and fractions were collected manually. Resulting fractions were dried using rotary evaporators. Resulting precipitates were dissolved in MeOH for antibacterial activity testing. Fractions from a given retention time that showed antibacterial activity were pooled from different HPLC runs and concentrated.

### Structure Elucidation of the Purified Compound

High-resolution mass analysis of the bioactive compound was performed using a MicrOTOF-QII mass spectrometer instrument (Bruker Daltonics GmbH, Germany).

^1^H and ^13^C-NMR spectra were recorded on a Bruker 600 Ascend spectrometer. Purified and lyophilized bioactive compound was dissolved in MeOH-*d_4_*. NMR spectra were referenced to residual solvent signals of methanol at δ_H_3.35 and δ_C_49.0. Coupling constants (*J*) are given in Hz. ^1^H and ^13^C-NMR spectroscopic assignment analyses were performed using correlation spectroscopy (COSY) and heteronuclear multi-bond correlation (HMBC) spectroscopy.

### LC-MS/MS Data Processing – Molecular Networking

Mass data were converted to mzXML format with Bruker’s Data Analysis software and uploaded to the Global Natural Product Social (GNPS) molecular networking tool^2^. In GNPS Wang et al. (2016), the data were subjected to molecular networking using the online workflow at GNPS. The data were clustered with MS-Cluster with a parent mass tolerance of 0.5 Da and a MS/MS fragment ion tolerance of 0.2 Da to create consensus spectra. A network was then created where edges parameters were cosine score above 0.2 and more than three matched peaks. The network was then searched against GNPS’s spectral libraries. The network analysis was exported from GNPS and analyzed in Cytoscape (Shannon et al., 2003).

## Results

### Soil Bacteria Isolation and Antibacterial Activities

In total 373 bacterial strains were isolated from rhizosphere samples of pseudo-savannah vegetation from three different arid regions in Tunisia. Thereunder, 25 isolates from Bou-Hedma, 140 from Chott-Djerid and 208 from Matmata. All strains were fast growing organisms and produced single colonies after 24h incubation at 37°C. Of these strains 29 (7.8%) showed moderate to strong antibacterial activity in the first screenings against *E. coli* ATCC 25922 and *S. aureus* ATCC 29213. These active strains were analyzed for their morphology and all bacteria showed a rod shape appearance. Gram-staining revealed that except for isolate C6, all isolates were Gram-positive bacteria. Ten of the 29 appeared non-motile, while the majority showed motility by microscopic investigation. The morphological characteristics (shape, margin, color, cell shape, motility, and Gram-staining) and the motility of the 29 soil bacteria are summarized in Supplementary Table 2.

To evaluate if the initial screening results could be verified, all strains were re-tested against a panel of Gram-positive and Gram-negative test strains; thereby all strains retained their activity (Supplementary Table 1). The most active strains were derived from all three sampling sites. Most interestingly, 93% were active against Gram-negative bacteria. Against Gram-positive bacteria very prominent activities were observed. Only strain C2 showed no activity against Gram-negative strains. Indeed, strain C2 showed the narrowest spectrum of activity, inhibiting solely the Gram-positives *S. aureus* ATCC 29213 and *S. epidermidis* (Supplementary Table 1). Four strains, i.e., C5, C6, C7, and C11, all from one sampling site, showed the largest activity spectrum with inhibition zones against all tested bacteria, except the clinical *E. coli* isolate.

### 16S rDNA Sequence Analysis

**FIGURE 2:**
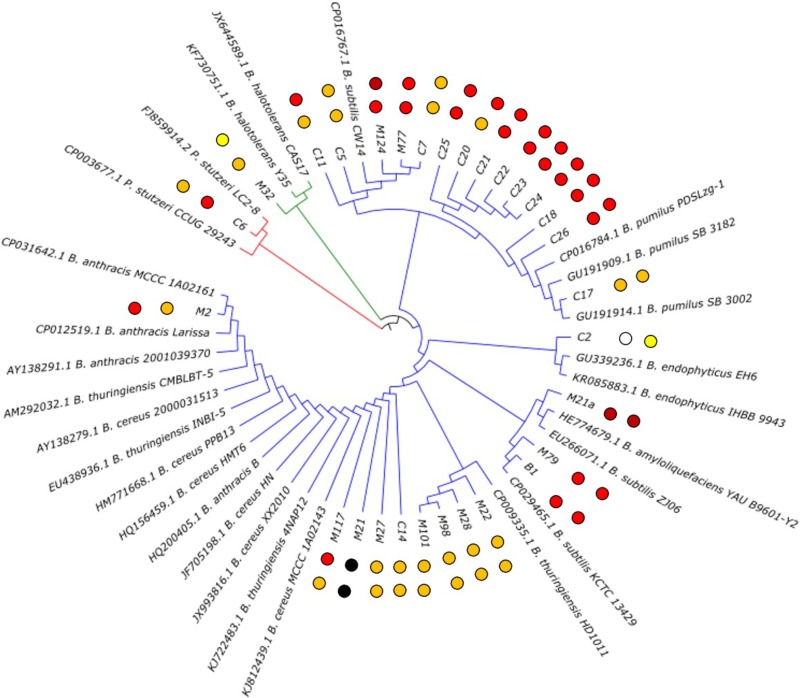
Phylogenetic tree of the isolated bioactive bacteria. The two closest homologoues based on 16 rDNA analysis for each isolate are given. The isolates from this study are carrying their identifier (compare Table 1). The observed antibacterial activity against *E. coli* ATCC25922 (inner dots) and *S. aureus* ATCC 29213 (outer dots) is indicated by dark red (strong activity), red (good activity), orange (moderate activity) and yellow (weak activity) dots. Strain M21 lost its viability during the project; therefore, the values could not be determined.

To obtain an insight into the diversity and phylogeny of the isolated strains showing antibiotic activity, these isolates were identified by 16S rRNA gene sequence analysis. The sequences obtained for the strains were compared to sequences deposited in publicly accessible databases. This revealed that the strains belong to three different bacterial phyla, i.e., *Firmicutes*, *Actinobacteria*, and *Proteobacteria*. However, *Bacillaceae* represented by far the most prominent family (86%), followed by *Brevibacteriaceae* (2%) and *Pseudomonadaceae* (2%). In fact, out of the 28 soil isolates, 26 were members of the genus *Bacillus*, one was *Brevibacterium halotolerans* and one was *Pseudomonas stutzeri* (Figure 2 and Table 1). Hence, in this project, strains of the genus *Bacillus* were found to dominate the strain collection. It seemed that they represent the most isolated bacterium from rhizospheric soil samples, which is in accordance with published data, since it was reported that the genus *Bacillus* is very common in soil by Amin et al. (2015). However, it must be considered that their ability to form highly resistant endospores is on the one hand the key for their successful colonization of a wide variety of environmental habitats, especially dry arid biological niches. On the other hand, the isolation strategy applied clearly favors spore formers.

**Table 1:** Closest relative of the 29 bioactive soil isolates based on 16S rDNA sequence.

Strain	Species^a^	Accession	Identity	Origin
B1	*Bacillus subtilis* KCTC 13429	CP029465.1	99	Bou-Hedma national park
C2	*Bacillus endophyticus* IHBB 9943	CP011974.1	98	Chott-Djerid
C5	*Bacillus subtilis* strain CW14	CP016767.1	99	Chott-Djerid
C6	*Pseudomonas stutzeri* strain LC2-8	FJ859914.2	99	Chott-Djerid
C7	*Bacillus subtilis* strain CW14	CP016767.1	99	Chott-Djerid
C11	*Bacillus subtilis* KCTC 13429	CP029465.1	99	Chott-Djerid
C14	*Bacillus cereus* strain XX2010	JX993816.1	99	Chott-Djerid
C17	*Bacillus pumilus* strain SB 3002	GU191914.1	99	Chott-Djerid
C18	*Bacillus pumilus* strain PDSLzg-1	CP016784.1	99	Chott-Djerid
C20	*Bacillus pumilus* strain PDSLzg-1	CP016784.1	99	Chott-Djerid
C21	*Bacillus pumilus* strain PDSLzg-1	CP016784.1	99	Chott-Djerid
C22	*Bacillus pumilus* strain PDSLzg-1	CP016784.1	99	Chott-Djerid
C23	*Bacillus pumilus* strain PDSLzg-1	CP016784.1	99	Chott-Djerid
C24	*Bacillus pumilus* strain PDSLzg-1	CP016784.1	99	Chott-Djerid
C25	*Bacillus pumilus* strain PDSLzg-1	CP016784.1	99	Chott-Djerid
C26	*Bacillus pumilus* strain PDSLzg-1	CP016784.1	99	Chott-Djerid
M2	*Bacillus anthracis* strain MCCC 1A02161	CP031642.1	99	Matmata
M21	*Bacillus thuringiensis* strain CMBLBT-5	AM292032.1	100	Matmata
M21a	*Bacillus subtilis* strain ZJ06	EU266071.1	99	Matmata
M22	*Bacillus thuringiensis* strain HD1011	CP009335.1	99	Matmata
M27	*Bacillus anthracis* strain B	HQ200405.1	99	Matmata
M28	*Bacillus anthracis* strain B	HQ200405.1	99	Matmata
M32	*Brevibacterium halotolerans* strain CAS17	JX644589.1	99	Matmata
M77	*Bacillus subtilis* strain CW14	CP016767.1	99	Matmata
M79	*Bacillus subtilis* KCTC 13429	CP029465.1	99	Matmata
M98	*Bacillus anthracis* strain MCCC 1A02161	CP031642.1	99	Matmata
M101	*Bacillus thuringiensis* strain HD1011	CP009335.1	99	Matmata
M117	*Bacillus thuringiensis* strain CMBLBT-5	AM292032.1	99	Matmata
M124	*Bacillus subtilis* strain CW14	CP016767.1	99	Matmata


*^a^The best hit obtained by BlastN analysis is given.*

### Isolation of 1-acetyl-β-carboline

#### Determination of the Fermentation Conditions

To get first insights into the metabolomic basis of the antibacterial activities observed, it was projected to isolate the underlying compound. First, a favorable culture medium was selected, to enable isolation of the antibacterial compound by high enough production yields. Therefore, strain M21a was cultured in 14 different media for 48 h at 30°C. Then the fermentation broth was tested by agar diffusion test against the test strains *E. coli* and *B. megaterium*. The composition of the culture media tested is given in Supplementary Table 3. All media selected promoted the growth of *Bacillus* sp. M21a, yielding visible biomass production. However, concerning the antibacterial activity, LB medium gave the best results against both, Gram-positive and Gram-negative test bacteria. In contrast, fermentation in M8 medium resulted in the lowest antibacterial activity observed. For all other 12 culture media, antibacterial activity was detected only against the Gram-positive test strain *B. megaterium* and not against the Gram-negative *E. coli*. Since we aimed to find the reason for the anti-Gram-negative activity, LB medium was chosen for the up-scaling.

#### Extraction, Purification and Structure Elucidation of 1-acetyl-β-carboline

For the isolation of the target compound, the producer strain *Bacillus* sp. M21a was fermented in 40 L LB medium. The organic phase of this fermentation broth was obtained by liquid-liquid extraction. The isolation of the compound was performed in a bioactivity-based manner. From the fermentation, 8 g dry weight were obtained and revealed a strong activity against *E. coli* (18 mm) and *B. megaterium* (30 mm) at a concentration of 5 mg/mL and 1 mg/mL, respectively. The bioactive crude extract was fractionated using Flash chromatography, which resulted in seven fractions. Subsequent bioactivity assay revealed the loss of activity against *E. coli*. However, one fraction, i.e., fraction 1, maintained activity against *B. megaterium*. For further fractionation, fraction 1 was separated by an analytical HPLC run. Three fractions had been collected and a 96 well plate assay was performed against the Gram-positive *Arthrobacter crystallopoietes*. We found that 200 μg of Fr1.1 showed a good antibacterial activity, whereby the other fractions showed no inhibition.

To finally purify the active compound, fraction Fr1.1 was purified by an additional HPLC separation. A peak with an adsorption maximum of 210 nm was observed at 12 min. This peak was manually collected and we obtained 1.8 mg of the bioactive compound.

**FIGURE 3:**
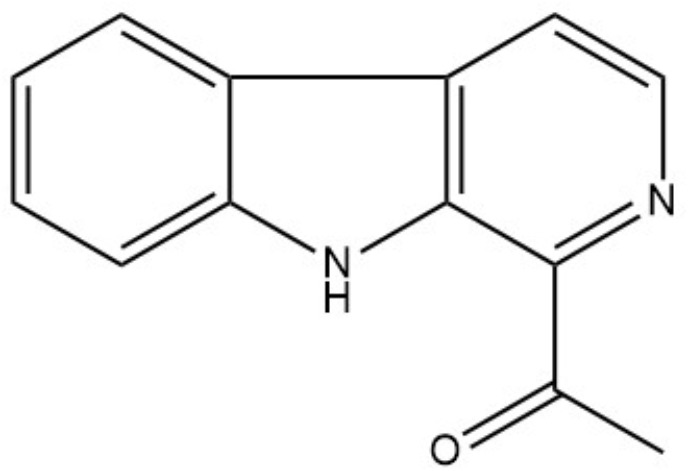
Structure of 1-acetyl-beta-carboline.

By high-resolution LC-MS analysis, the mass was determined to be *m/z* 211.0907 [M + H]^+^ (Supplementary Figure 1). This mass fitted to a compound with the molecular formula C_13_H_10_N_2_O (calculated *m/z* for [M + H]^+^: 211.0871). To identify the bioactive compound unambiguously, NMR analyses were performed. The ^1^H-NMR spectrum revealed signals for six aromatic protons (δ_H_7.32; 7.61; 7.71; 8.23; 8.47; and 8.32), two ortho-coupled doublets at δ_H_7.71 and 8.23 (*J* = 7.9 Hz) and four multiplet protons of which two were triplets at δ_H_7.32 and 7.61 (*J* = 7.9 Hz) (Supplementary Table 4 and Supplementary Figure 2). These data indicated the presence of a 1,2-disubstituted aromatic ring. In the aliphatic region, the ^1^H-NMR spectrum showed the presence of an aromatic bounded methyl group singlet at δ_H_2.83. The ^13^C-NMR spectrum showed signals for 13 carbon atoms (Supplementary Figure 3). Among these, six aromatic methine *C*-atoms (δ_C_129.8; 130.2; 113.4; 122.5; 138.54; and 120.1) and five sp^2^ quaternary carbons (δ_C_143.3; 121.5; 132.3; 130.7; and 137.1) (Tab. S4). The remaining two carbons were considered as an acetyl group attached to C-9, indicated by the HMBC correlation between H-13 and C-9. The COSY (Supplementary Figure 4) and HMBC data (Supplementary Figure 5) led to reveal the structure of 1-acetyl-β-carboline (Figure 3) that was confirmed by the comparison with literature data (Lee et al., 2013).

**FIGURE 4:**
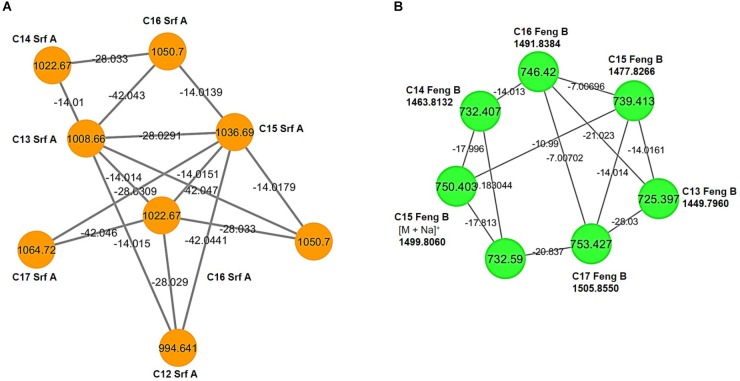
Molecular networks of *Bacillus* sp. M21a extracts. **(A)** Surfactin A homologs ([M + H]^+^; orange), the *m/z* differences of 14.01, 28.03, and 42.04 indicate molecules with different lengths of fatty acid chains within this cluster. **(B)** Fengycin B cluster from double charged precursor ions *m/z* ([M + 2H]^2+^; green). Nodes represent the precursor ion and bridges indicate the mass difference between individuals nodes. Labels at the nodes name the respective dereplicated molecule. Srf, surfactin; Feng, fengycin, the number indicates the length of the lipid tail.

### Identification of Bioactive Lipopeptides

In addition to the here isolated carboline, derivatives of the known fungicides bacillomycin and fengycin were identified by mass-based analyses (Figure 4). These bioactive lipopeptides from the methanolic extract of a solid cultivation of *Bacillus* sp. M21a were detected using LC/MS in positive ion mode. In the MS spectra, three clusters of molecular mass ions belonging to the iturin, surfactin and fengycin lipopeptide families were found. These included four bacillomycins, members of the iturin family, having fatty acyl chain lengths of C13–C16, three fengycins A (from C14 to C16 and three fengycins B (C15–C17), and six surfactins (C12–C17).

#### Bacillomycin Identification

The protonated molecular mass ions [M + H]^+^ at m/z 1017.51, 1031.52, 1045.53, and 1059.55 (Supplementary Figure 3A) were assigned as putative homologs of bacillomycin D, a variant of the iturin molecule group, with C13, C14, C15, and C16 β-amino fatty acids, respectively (Cao et al., 2012). On the basis of NORINE’s database, which contains the most common non-ribosomal peptides, these compounds were identified as C13-bamD (C_47_H_72_N_10_O_15_), C14-bamD (C_48_H_74_N_10_O_15_), C15-bamD (C_49_H_76_N_10_O_15_), and C16-bamD (C_50_H_78_N_10_O_15_). The sequence of the compound C14-Bam D with an *m/z* of 1,031.5 was determined from series of *N*-terminal and proline-directed fragments (Supplementary Figure 4A). The peptide ring of this compound was cleaved both at the peptide bond between its amino fatty acid residue (β-NH_2_-FA) and threonine at position 7 (Thr_7_) as well as at the N-terminal of proline 4 (Pro_4_). In the first case, fragment ions at *m/z* 340.2, 504.2, and 617.3 were detected and corresponded to [M + H-Tyr-Asn-Pro-Glu-Ser-Thr]^+^, [M + H-Asn-Pro-Glu-Ser-Thr]^+^, and [M + H-Pro-Glu-Ser-Thr]^+^, respectively. In the second case, fragment ions at *m/z* 227.1, 314.1, 441.3, 617.3, 754.4, and 917.4 were observed and corresponded to [M + H-Ser-Thr-FA-Asn-Tyr-Asn]^+^, [M + H-Thr-FA-Asn-Tyr-Asn]^+^, [M + H-FA-Asn-Tyr-Asn]^+^, [M + H-Asn-Tyr-Asn]^+^, [M + H-Asn-Tyr]^+^, and [M + H-Asn]^+^, respectively. The MS/MS spectrum of [M + H] ^+^ ion at *m/z* 1045.5 presented fragments with *m/z* at 517.3, 618.3, 654.4, 732.4, 768.4, 829.4, 931.5, and 958.5. The corresponding amino acid sequence is given in Supplementary Figure 4B. In lower intensity *Bacillus* sp. M21a produced C16-Bam D and its fragmentation pattern is given in Supplementary Figure 4C.

#### Fengycin Identification

The LC-MS data and the molecular networking analysis showed that the ions corresponding to fengycins were [M + 2H]^+^ (*m/z* 718.392) for C14-fenA (C_70_H_106_N_12_O_20_), [M + 2H] ^+^(*m/z* 725.397) for C15-fenA (C_71_H_108_N_12_O_20_), [M + 2H] ^+^ (*m/z* 732.407) for C16-fenA (C_72_H_110_N_12_O_19_), [M + 2H] ^+^ (*m/z* 739.413) for C15-fenB (C_72_H_110_N_12_O_20_), [M + 2H] ^+^ (*m/z* 746.42) for C16-fenB (C_74_H_114_N_12_O_20_), and [M + 2H] ^+^ (*m/z* 753.427) for C17-fenB (C_75_H_116_N_12_O_20_) (Supplementary Figure 3B). The fragmentation patterns of C15-FenA, C16-FenA, C15-FenB and C17-FenB are given in Supplementary Figure 5.

#### Surfactin Identification

The isolate M21a was also able to produce surfactins. Ion peaks at *m/z* 994.64, 1008.65, 1022.67, 1036.68, 1050.69, and 1064.71 were obtained and correspond to C12 to C17-srf, respectively (Supplementary Figure 3C). The MS/MS spectra of surfactin isoforms showed common fragments [M + H]^+^ at *m/z* 685.45, 671.43, and 699.46 (Supplementary Figure 6). These ions are from characteristic amino acid sequences, previously reported as Val/Leu/Asp/Val/Leu/Leu, Leu/Leu/Asp/Val/ Leu/Leu, Leu/Leu/Asp-OMe/Val/Leu/Leu and Leu/Leu/Asp/Leu/Leu/Leu, respectively (Bonmatin et al., 1995; Tang et al., 2010; Biniarz and Lukaszewicz, 2017). The MS/MS spectrum of [M + H]^+^ ion at *m/z* 1036.6869 presented fragments corresponding to losses of amino acids Leu/Leu/Asp/Val/Leu/Leu, with *m/z* 923.6029, 810.5212, 695.4953, 596.4275, 483.3442, and 370.2595, respectively (Supplementary Figure 4). The *m/z* 370.2595 corresponded to glutamic acid residue with aliphatic fatty acid chains containing 15 carbons, indicating similarities between the proposed surfactin A (C15) produced by *Bacillus* sp. M21a and the known surfactin (Liao et al., 2016). Additional fragment ions [M + H]^+^ confirmed the presence of amino acids sequence as *m/*z 227.1766 [Leu + Leu + H] ^+^, *m/z* 328.1873 [Leu + Asp + Val + H] ^+^, *m/z* 441.2721 [Leu + Asp + Val + Leu + H]^+^, and *m/z* 554.3560 [Leu + Asp + Val + Leu + Leu + H] ^+^ (Supplementary Figure 5). The molecular networking analysis showed compounds with *m/z* differences of 14.01, 28.03, and 42.04, suggesting molecules with different lengths of fatty acid chains within the same isoform family (Figure 4). Surfactin isoforms [M + H]^+^ are displayed in Table 2.

**Table 2:** Lipopeptide identified in *Bacillus* sp. M21a.

Structure	Molecular formula	[M + H] ^+^	Observed [M + H] ^+^
**BACILLOMYCIN D**			
C13	C_ 47_H_72_N_10_O_15_	1017.5000	1017.5102
C14	C_ 48_H_74_N_10_O_15_	1031.5395	1031.5245
C15	C_ 49_H_76_N_10_O_15_	1045.5551	1045.5382
C16	C_ 50_H_78_N_10_O_15_	1059.5707	1059.5520
C17	C_ 51_H_81_N_10_O_15_	1073.5863	1073.5680
**FENGYCIN**			
C14 – Fen A	C_70_H_106_N_12_O_20_	1435.7725	1435.7854
C15 – Fen A	C_71_H_108_N_12_O_20_	1449.7881	1449.7960
C16 – Fen A	C_72_H_110_N_12_O_20_	1463.8038	1463.8132
C15 – Fen B	C_72_H_110_N_12_O_20_	1477.8194	1477.8266
C16 – Fen B	C_74_H_114_N_12_O_20_	1491.8351	1491.8384
C17 – Fen B	C_75_H_116_N_12_O_20_	1505.8510	1505.8550
**SURFACTIN A**			
C12	C_ 50_H_87_N_7_O_13_	994.6440	994.6401
C13	C_ 51_H_89_N_7_O_13_	1008.6597	1008.6550
C14	C_ 52_H_91_N_7_O_13_	1022.6753	1022.6701
C15	C_ 53_H_93_N_7_O_13_	1036.6912	1036.6843
C16	C_ 54_H_95_N_7_O_13_	1050.7059	1050.6999
C17	C_ 55_H_97_N_7_O_13_	1078.7379	1064.7100


## Discussion

The development of effective medicinal drugs has been revolutionizing the treatment of many human diseases. Lead structures for these drugs were detected by natural product research, since it seems that nature represents a most important resource for biologically active compounds. However, concerning antibiotics, which can be regarded as option of choice to combat bacterial infections, most of the drugs in application were already discovered several decades ago (Pidot et al., 2014). The increase of highly resistant pathogenic bacteria could put us back into a situation comparable to the pre-antibiotic era. Thus, there is an urgent need for a new age of antibiotic discovery (Khan and Khan, 2016). Ninety percent of all antibiotics, which are in clinical use today, are derived from microorganisms (Katz and Baltz, 2016) and the majority of the modern classes of antibiotics was discovered by antimicrobial activity-based screening approaches of microorganisms isolated and cultured predominantly from soil (Rolain et al., 2016). In other words, bacteria have so far been and can be also expected to remain the most promising resource for antibiotics in the future, since the majority of drugs has been developed from lead structures on the basis of bacterial natural products (Elbandary et al., 2018). Proven proliferative antibiotic-producing bacterial species include Actinomycetes, predominantly the genus *Streptomyces* (Hug et al., 2018), myxobacteria (Mulwa et al., 2018), cyanobacteria (Senhorinho et al., 2015), *Bacilli* (Sumi et al., 2015), and Pseudomonads (Gross and Loper, 2009). Even though many compounds have been already isolated from the before mentioned bacteria, it must be considered that uncultured bacteria sum up to over 99% of all species in external environments (Ling et al., 2015). Hence, there could still be a multitude of metabolites awaiting discovery, and even for well-studied producer organisms it was shown that they harbor many more biosynthetic gene clusters (BGC) for the production of specialized metabolites than natural products described from them (Pidot et al., 2014).

Only a few reported surveys exist on the bacterial diversity in arid areas, where bacterial species should be able to deal with relatively high temperatures, salt concentrations, and radiation. Hence, arid and desert habitats represent special ecosystems, and it can be expected that the chance to isolate uncommon bacteria with new metabolic capabilities is high (Trabelsi et al., 2016). In the present study, bacteria were isolated from Tunisian underexplored arid areas. Thereunder, 28 rhizospheric bacteria showed the ability to produce natural products effective against both Gram-positive and Gram-negative bacteria. The majority, 26 isolates, belonged to the Firmicutes phylum (genus *Bacillus*); one isolate belonged to the Actinobacteria phylum (genus *Brevibacterium*) and one to the Proteobacteria phylum (genus *Pseudomonas*). This is in accordance to the fact that *Bacillus* species are dominant soil bacteria. Their abilities of endospore-formation and antibiotics production have to be regarded as an advantage for the colonization of environments (Moshafi et al., 2011; Amin et al., 2015). Antimicrobial peptides of *Bacilli* were regarded as a promising starting point in the search for new antibacterial drugs (Sumi et al., 2015), and several known antibacterial compounds had been isolated, e.g., subtilin (Kim et al., 2012), surfactin (Jacques, 2011), and macrolactin A (Lu et al., 2008). Even though in this screening only one actinobacterial strain was isolated, they have been shown to be a resource with an unprecedented diversity of biosynthetic pathways, awaiting to be exploited for the identification of novel scaffolds. The latter could then contribute to the filling of the antibiotics development pipeline. *Brevibacterium halotolerans* SA87, a close homolog to the here isolated strain, has been shown to produce a variety of specialized metabolites with distinct bioactivities. It was shown that *B. halotolerans* SA87inhibits the growth of both Gram-positive and Gram-negative pathogens (Ahmed et al., 2015). Concerning *Pseudomonas* species, many specialized metabolites are reported and reviewed (Gross and Loper, 2009). This group of bacteria adapts to different stress environments and produces a wide range of bioactive metabolites with antimicrobial activity and various *Pseudomonas* species are able to biosynthesize compounds with a broad biological activity (Robles-Huízar et al., 2017). *Pseudomonas stutzeri* CMG1030, the closest homolog to strain C6, was reported to biosynthesize zafrin, an antibacterial compound, which showed strong antibacterial activity against Gram-positive as well as against Gram-negative bacteria (Uzair et al., 2008).

The strain *Bacillus* sp. M21a showed strong antimicrobial activity against Gram-positive and Gram-negative test strains and was chosen for further investigation and isolation of the bioactive compound. However, after fractionation the activity against *E. coli* was lost. This might be due degradation of the active compound(s), or to synergistic effects between different compounds, which were separated by the fractionation. The Gram-positive active compound was identified as 1-acetyl-β-carboline. The structure of this compound was unambiguously proven by NMR experiments and the data obtained were in good agreement with reported literature values (Huang et al., 2011; Lee et al., 2013). This compound was previously isolated from several types of organisms. Thereunder plants like *Ailanthus malabarica* and *Hypodematiumsquamuloso-pilosum* (Joshi et al., 1977; Zhou et al., 1998), a fungus *Neosartorya pseudofischeri* (Lan et al., 2016), a sponge *Tedaniaignis* (Dillman and Cardellina, 1991), as well as several bacteria, e.g., *Steptomyces* and *Pseudomonas* spp. (Proksa et al., 1990; Shin et al., 2010; Huang et al., 2011; Chen et al., 2013; Lee et al., 2013; Broberg et al., 2017). The β-carboline alkaloid displays various biological activities, e.g., antitumor, antimicrobial, antiviral, and antiparasitic (Cao et al., 2007; Lee et al., 2013). 1-acetyl-β-carboline showed antibacterial activity against both, MSSA and MRSA strains. The reported activities have to be considered as moderate, however, the MRSA strain tested was inhibited more efficiently by 1-acetyl-β-carboline (MICs in the range of 32–128 μg/mL) than by the β-lactams tested (ampicillin, penicillin and oxacillin, MICs in the range of 64–512 μg/mL). Further, 1-acetyl-β-carboline was described to possess synergistic effects with ampicillin and penicillin. Shin et al. (2010) found that the fractional inhibitory concentration (FIC) indices of 1-acetyl-β-carboline in combination with ampicillin and of 1-acetyl-β-carboline in combination with penicillin were in the range of 0.156–0.313 and of 0.188–0.375 in combination with 32 and 64 μg/mL of 1-acetyl-β-carboline against the MRSA strains tested. Thereby, a synergistic effect was observed. However, it did not exhibit a synergistic antibacterial effect against most of the MRSA strains tested, if combined with oxacillin. The same difference in synergistic effects has been also observed in combinations of dieckol and β-lactams (Lee et al., 2008), as well as epigallocatechin gallatein combination with β-lactams (Zhao et al., 2001). The mechanism of the observed synergism between 1-acetyl-β-carboline and β-lactams is unknown. It was speculated that a possible reason for the synergistic effect can be attributed to the fact that both molecules attack the same target of the cell wall, but on different sites due to their different structures (Shin et al., 2010). Another study also reported that 1-acetyl-β-carboline in contrast to β-lactams was active against both MRSA and MSSA (Lee et al., 2013). In light of these findings, it seems reasonable that the antibacterial activity of 1-acetyl-β-carboline is not related to PBP2, the main target site of β-lactams in the MSSA cell wall, since it was also active against MRSA cells, which have PBP2a in the cell wall. The latter has a low affinity toward β-lactams. The biosynthesis of 1-acetyl-β-carboline was described for *Marinactinospora thermotolerans*. In this bacterium, a BGC, consisting of the three genes *mcbA, mcbB* and *mcbC*, was identified to be responsible for the generation of the β-carboline scaffold. This scaffold is generated by a Pictet-Spengler (PS) reaction, using tryptophan (or tryptamine) and an aldehyde as substrates. The PS reaction is an important reaction for the synthesis of natural products especially in those bearing indole and isoquinoline alkaloids as scaffolds in their complex structures (Heravi et al., 2018). The novel enzyme McbB encoded in this BGC was characterized to catalyze the PS cyclization/decarboxylation/oxidation process. By heterologous expression experiments in *Streptomyces lividans* TK64 and *Escherichia coli* BL21, it was found that *mcbB* was sufficient in *E. coli* for the production of 1-acetyl-3-carboxy-β-carboline, which represents the major product. Furthermore, two minor products, i.e., 1-acetyl-β-carboline and 1-acetyl-3-hydroxy-β-carboline, are generated (Chen et al., 2013). By *in vivo* gene inactivation and bioinformatic tools, Chen et al. (2013) assigned the other proteins encoded in the BGC as a CoA ligase (McbA) and a decarboxylase (McbC).

It was reported before that 1-acetyl-β-carboline produced by *Streptomyces* sp. 04DH52 exhibits only a moderate antibacterial activity against Gram-negative bacteria with MICs ranging from 64 to 256 μg/mL (Shin et al., 2010). Also Lee et al. (2013) reported that 1-acetyl-β-carboline, isolated from *Pseudomonas* sp. UJ-6, showed antibacterial activity against Gram-negative bacteria with MICs ranging from 32 to 128 μg/mL. However, all the reported activities are in the moderate to low range. In our hands, *Bacillus* sp. M21a extracts lost *E. coli* activity after fractionation by Flash chromatography. This can be explained by a number of possible reasons. When we tested the organic phase of *Bacillus* sp. M21, many compounds were present in this mixture and might be interacting with one another to result in the inhibition observed. When the compounds were separated by using Flash chromatography, these could not interact anymore, which resulted in the loss of *E. coli* inhibition. It can be speculated that the compound 1-acetyl-beta-carboline was likely working with an unkown compound to yield the inhibitory effect against *E. coli*. Separation of the compounds abolished the synergistic effects they had in combination. Another reason why this occurred might be the instability of the active component during the Flash fractionation and dryness process, due to light or high temperature sensitivity leading to changes in the conformation of the active molecule, before testing the activity again. Another factor could have accounted for such a loss of activity; it might be a question of concentration that means that after fractionation we lost an amount of the active compound and its concentration, in one of the collected fractions, wasn’t sufficient to inhibit the test *E. coli* strain.

In addition to the antibacterial compound, further fungicides were identified. These natural products are involved in the plant growth promoting effects described for various *Bacilli*. Fengycins, composed of 10 amino acids linked to a fatty acid chain with 14–19 carbon atoms, exhibit a strong antifungal activity due to their interaction with the cell membrane, which results in an increased cell permeability, finally leading to an ultrastructural destruction of pathogenic fungi (Yang et al., 2015). Fengycins were described to possess an antibacterial activity against the Gram-positive *S. epidermidis* and the Gram negative *E. coli* (Huang et al., 2006; Roy et al., 2013). Huang et al. (2006) reported also that fengycins showed an antiviral activity against Newcastle disease virus and bursal disease virus. An antitumoral activity has also been attributed to this decalipopeptide (Sivapathasekaran et al., 2010; Yin et al., 2013; Ditmer, 2014). The inhibition of plant fungal pathogens by bacillomycin D and fengycins, was shown to be based on the induction of ROS production (Tang et al., 2014; Han et al., 2015; Gu et al., 2017). The bacillomycin D analogs have been mostly assayed for their antifungal activities (Tabbene et al., 2016), especially against phytopathogenic fungi (Moyne et al., 2001; Tanaka et al., 2014; Kim et al., 2016). Recently, bacillomycin D has showed antitumoral activities (Hajare et al., 2013; Gong et al., 2014; Qian et al., 2015; Sun et al., 2018). Surfactin possesses a number of biological activities. It is known for its antiviral, antibacterial and antitumoral properties (Meena and Kanwar, 2015; Moro et al., 2018).

## Conclusion

It was shown that the strain collection of rhizospheric bacteria from Tunisian arid areas has a potential for the discovery of compounds with antibacterial and antifungal activities. Bacillomycin D, fengycin A and B, and surfactin were identified based on LC-MS data and molecular networking analysis. In addition, 1-acetyl-β-carboline was isolated as pure compound, which is known to have synergistic effects with other antibiotics. Overall, the strain collection is dominated by the Gram-positive genus *Bacillus*, which seems to play an important role in shaping the microbiome by the production of bioactive compounds.

## Author Contributions

GK, TS, and RB designed and planned the research. ZN isolated and identified the bacterial strains. ZN and HB isolated natural products. SK guided laboratory work and analyzed the NMR data. All authors analyzed and interpreted the results and commented on the manuscript prepared by ZN and TS.

## Conflict of Interest Statement

The authors declare that the research was conducted in the absence of any commercial or financial relationships that could be construed as a potential conflict of interest.

## Footnotes

1 http://www.ncbi.nlm.nih.gov/

2 https://gnps.ucsd.edu/ProteoSAFe/static/gnps-splash.jsp
