# Supplementary material for: Soil Bacteria Isolated From Tunisian Arid Areas Show Promising Antimicrobial Activities Against Gram-Negatives
Source: Front Microbiol. 2018 Nov 13;9:2742. doi: 10.3389/fmicb.2018.02742 (PMC6242944; doi:10.3389/fmicb.2018.02742)
Supplement: Supplementary file 1 [file Data_Sheet_1.docx]

Supplementary Material

Soil Bacteria Isolated from Tunisian Arid Areas Show Promising Antimicrobial Activities against Gram-Negatives

Zina Nasfi^1,2,3^, Henrik Busch^4^, Stefan Kehraus^4^, Luis Linares-Otoya^2^, Gabriele M. König^4^, Till F. Schäberle^2,5*^, Rafik Bachoual^1^

^1^Laboratory of Plant Improvement and Valorization of Agroresources, National School of Engineering of Sfax, Sfax, Tunisia

^2^Institute for Insect Biotechnology, Justus-Liebig-University Giessen, Giessen, Germany

^3^Faculty of Sciences of Gabès, University of Gabès, Tunisia

^4^Institute for Pharmaceutical Biology, University of Bonn, Bonn, Germany

^5^Department of Bioresources of the Fraunhofer Institute for Molecular Biology and Applied Ecology, Giessen, Germany

*** Correspondence:**
till.f.schaeberle@agrar.uni-giessen.de

# Supplementary Figures and Tables

- 1. **Supplementary Figures**

##
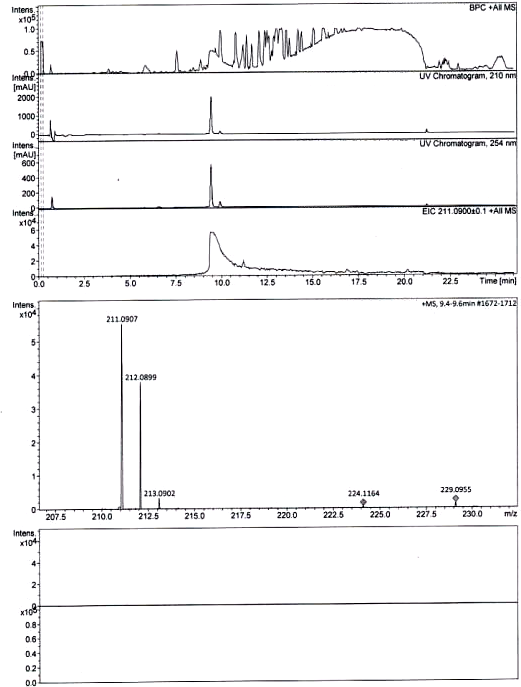


##

**Supplementary Figure 1.** LC-MS mass spectrum of the bioactive compound 1-acetyl-β-carboline produced by *Bacillus* sp. M21a.

(A)

**
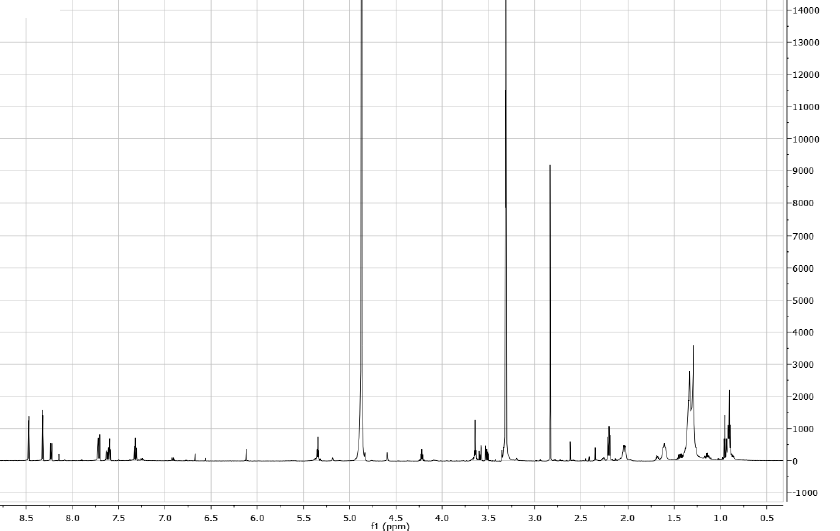
**

(B)

**
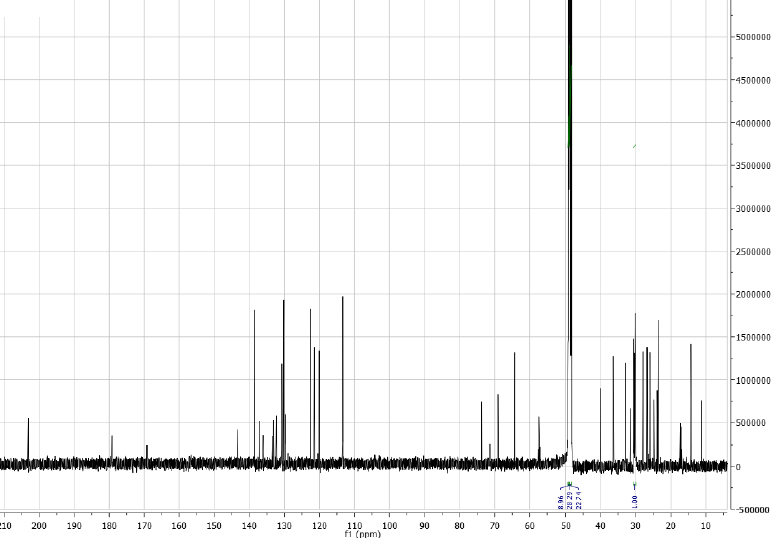
**

(C)

**
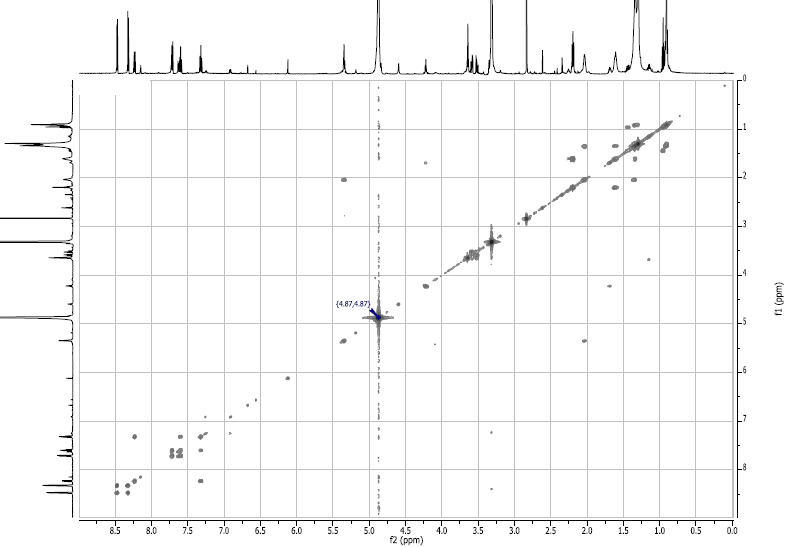
**

(D)

**
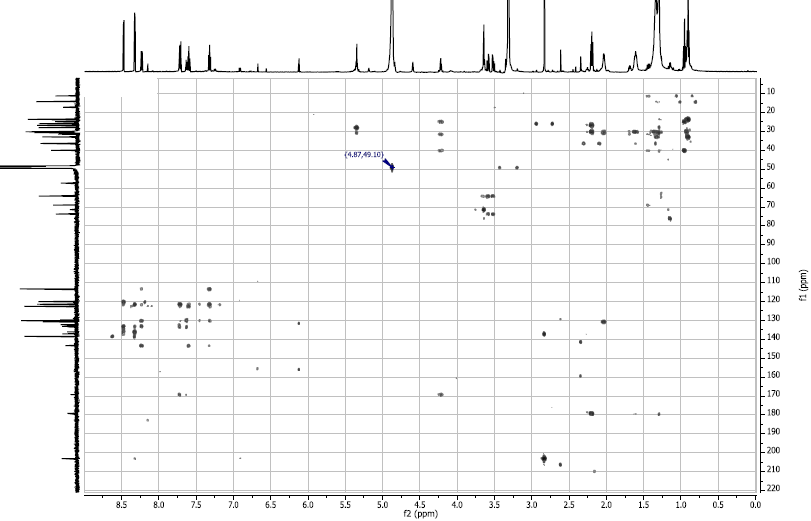
**

**Supplementary Figure 2.** NMR data of the bioactive compound 1-acetyl-β-carboline produced by *Bacillus* sp. M21a. (A) ^1^H-NMR spectrum, (B) ^13^C-NMR spectrum, (C) ^1^H-^1^H COSY spectrum, (D)^1^H-^13^C HMBC spectrum.

(A)


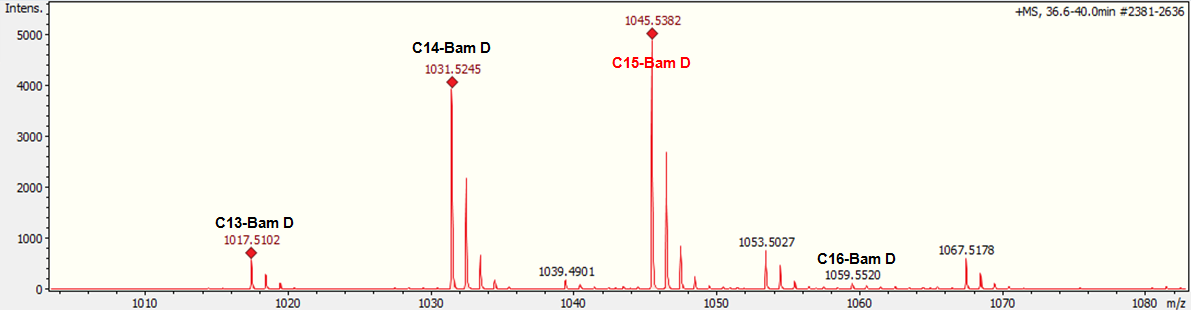


(B)


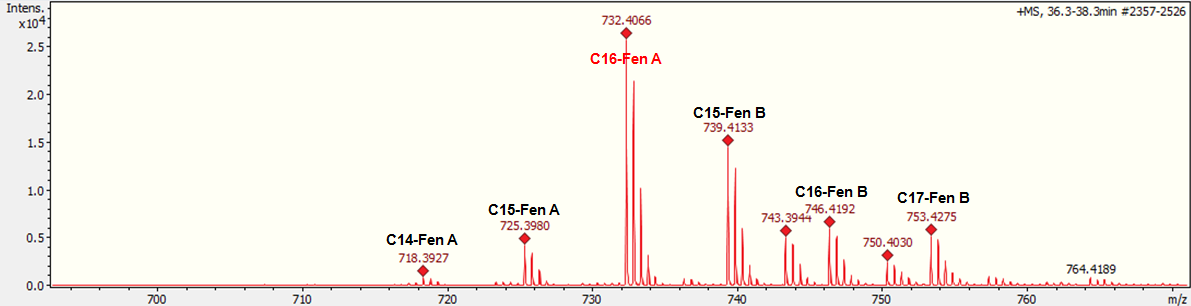


(C)


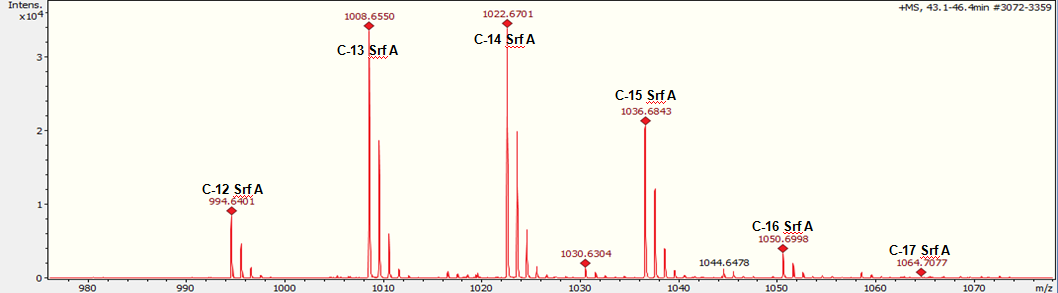


**Supplementary Figure 3.** LC-MS spectra of bioactive lipopetides from the methanolic extract from a solid cultivation of *Bacillus* sp. M21a. (A) Mass spectra corresponding to the bacillomycin D homolog, (B) Mass spectra corresponding to the fengycin homolog, (C) Mass spectra corresponding to the surfactin homolog.


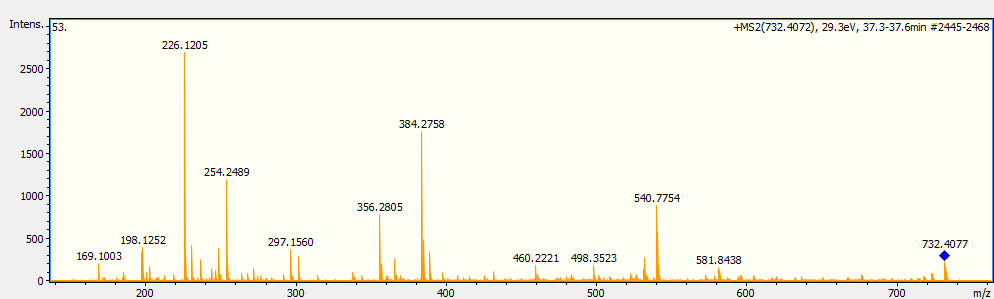

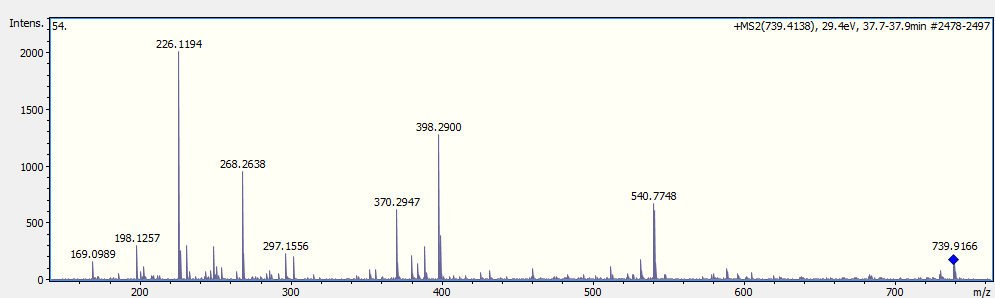

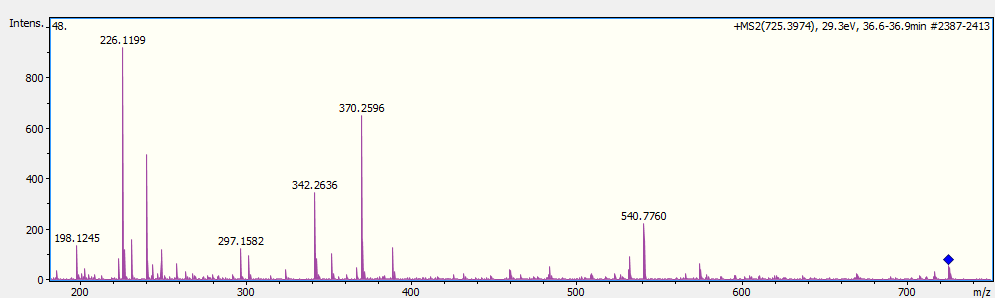

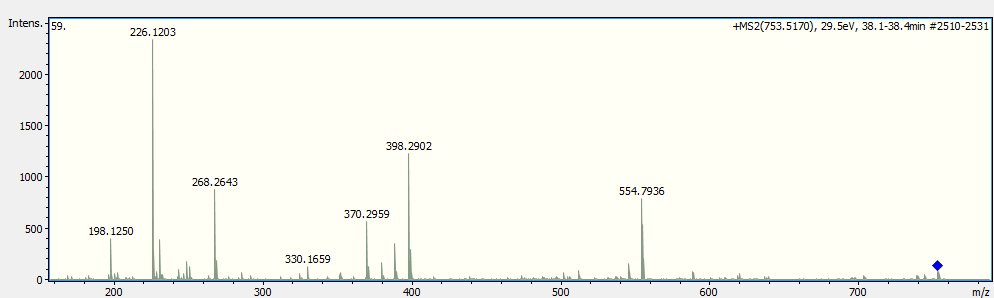


**Supplementary Figure 4.** MS/MS spectrum of the fengycin precursors, **A** C15-Fen A precursor ion [M + 2H]^2+^ at *m/z* 725.3974, **B** C16-Fen A precursor ion [M + 2H]^2+^ at *m/z* 732.4072, **C** C15-Fen B precursor ion [M + 2H]^2+^ at *m/z* 739.4138, **D** C17-Fen B precursor ion [M + 2H]^2+^ at *m/z* 753.5170

(A)

**
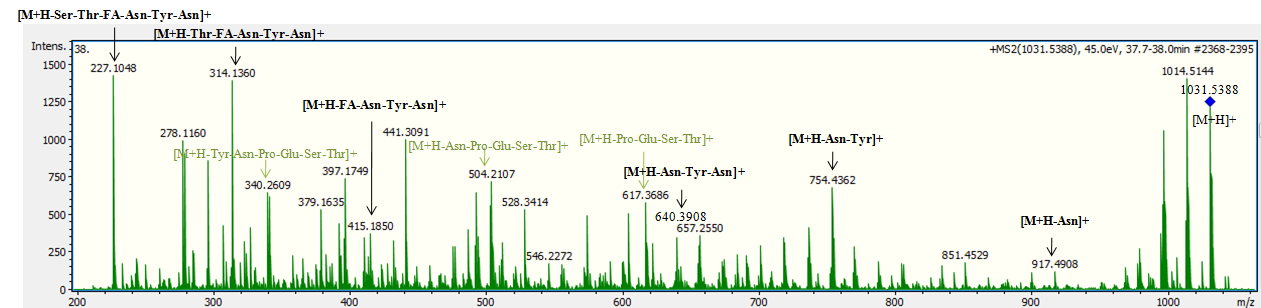
**

(B)

**
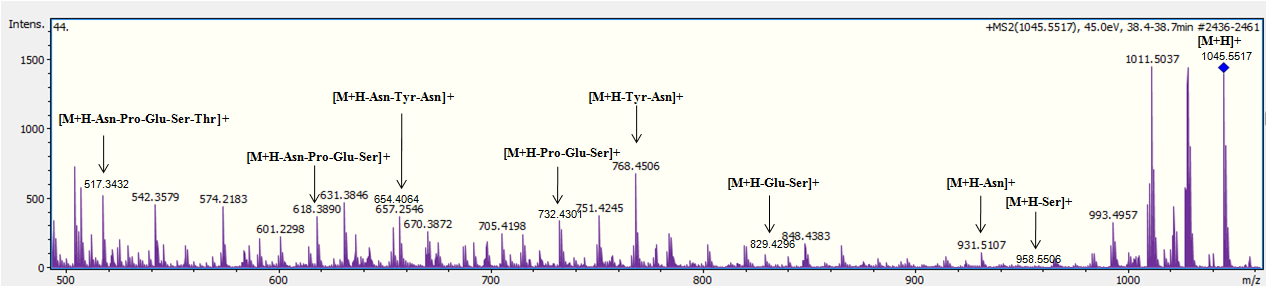
**

(C)

**
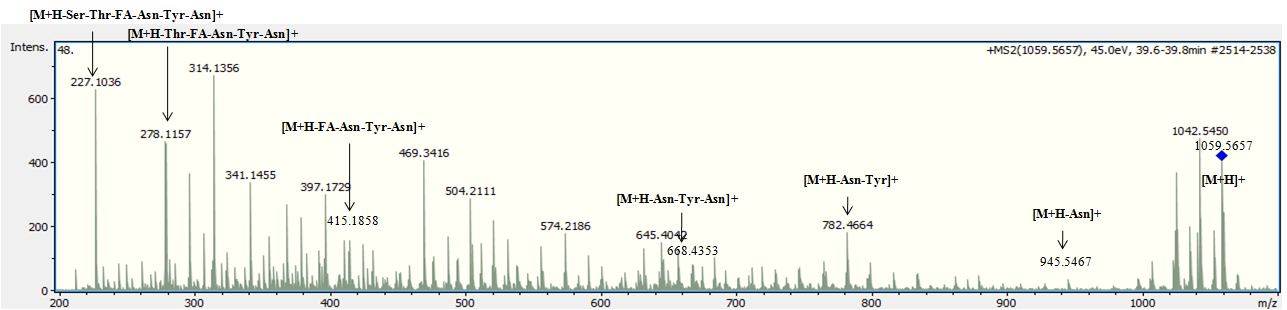
**

**Supplementary Figure 5.** MS/MS spectrum of the bacillomycin D precursors, **A** Bam D precursor ion [M + H]^+^ at *m/z* 1031 for C14-Bam D, **B** Bam D precursor ion [M + H]^+^ at *m/z* 1045 for C15-Bam D, **C** Bam D precursor ion [M + H]^+^ at *m/z* 1059 for C16-Bam D.

(A)
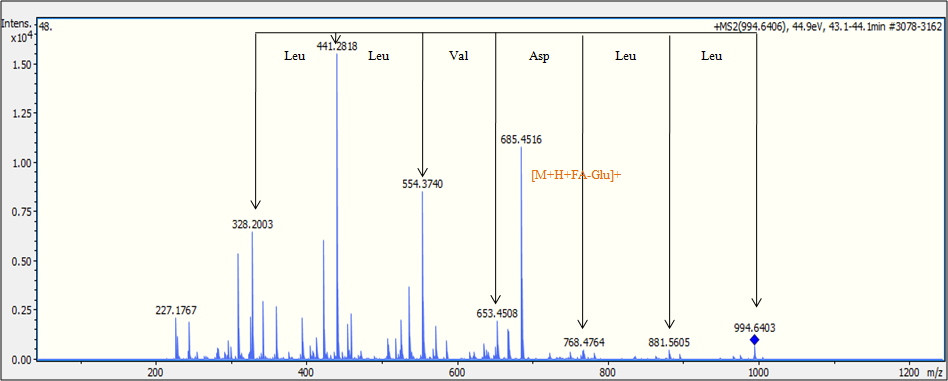


(B)
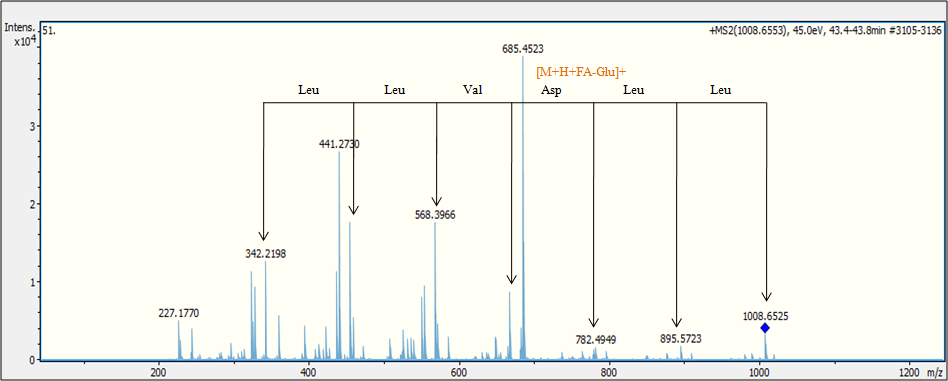


(C)
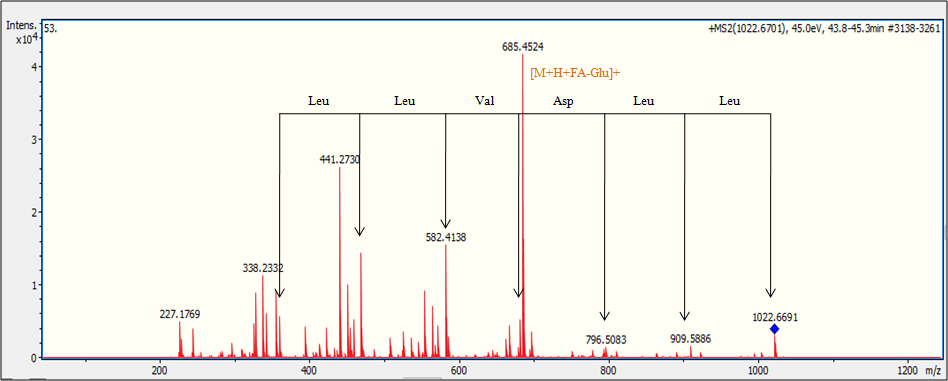


(D)
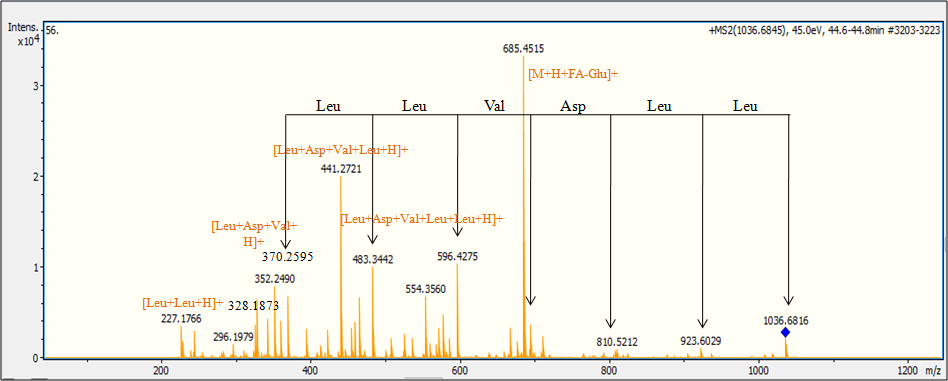


(E)
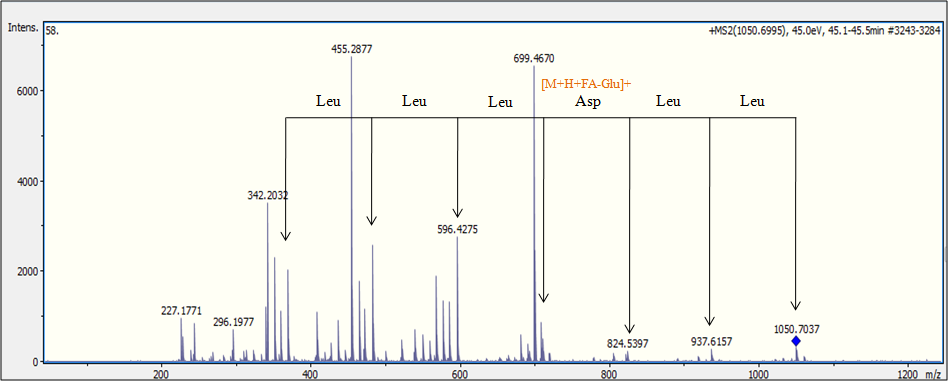


(F)
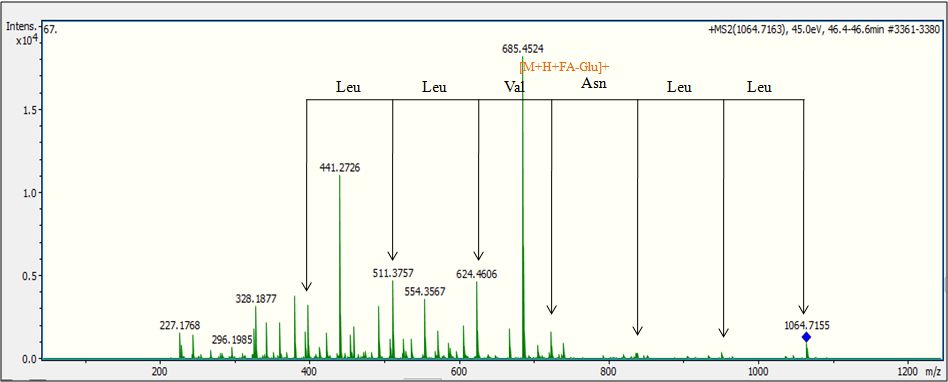


**Supplementary Figure 6.** MS/MS spectrum of the surfactin precursors and fragmentation proposal, **A** Surfactin precursor ion [M + H]^+^ at *m/z* 994 for C12-Srf A, **B** Surfactin precursor ion [M + H]^+^ at *m/z* 1008 for C13-Srf A, **C** Surfactin precursors ion [M + H]^+^ at *m/z* 1022 for C14-Srf A, **D** Surfactin precursor ion [M + H]^+^ at *m/z* 1036 for C15-Srf A, **E** Surfactin precursor ion [M + H]^+^ at *m/z* 1050 for C16-Srf A, and **F** Surfactin precursor ion [M + H]^+^ at *m/z* 1064 for C17-Srf A

## Supplementary Tables

**Supplementary Table1.** Antibacterial activity of the selected soil isolates against several test bacteria.

|  | **Gram Negative Bacteria** | | | | | | | | | | | | | | | **Gram Positive Bacteria** | | | | | | |  |
| --- | --- | --- | --- | --- | --- | --- | --- | --- | --- | --- | --- | --- | --- | --- | --- | --- | --- | --- | --- | --- | --- | --- | --- |
| Strain | ***E. coli* ATCC25922** | ***E. coli* ATCC35218** | ***E. coli* KL.16** | ***E. coli* KL16.2a** | | ***E. coli* MA isolate** | | ***E. coli* isolate** | | ***S. typhi*isolate** | | ***S. typhi*isolate** | | ***S aureus* ATCC29213** | | | ***S. epidermidis*** | | ***S. saprophyticus*** | | ***S. aureus* isolate** | |  |
| **B1** | 13,33 ± 1,15 | 14 ± 0 | 12,33 ± 0,57 | 11,66 ± 0,57 | | 10 ± 0 | | - | | ND | | ND | | 19,66 ± 2,08 | | | 19 ± 1 | | 15,33 ± 0,57 | | - | |  |
| **C2** | - | - | - | - | | - | | - | | - | | - | | 9 ± 0 | | | 8,33 ± 0,57 | | - | | - | |  |
| **C5** | 13 ± 0,57 | 12,66 ± 1,52 | 11,66 ± 0,57 | 11,66 ± 1,15 | | 9 ± 0 | | - | | 16,33 ± 0,57 | | 16 ± 0 | | 12,66 ± 0,57 | | | 12,66 ± 1,52 | | 13,66 ± 1,52 | | - | |  |
| **C6** | 14,33 ± 0,57 | 15,33 ± 0,57 | 15 ± 0 | 14 ± 0 | | 12,33 ± 0,57 | | - | | 17 ± 1,73 | | 16,33 ± 1,15 | | 12,66 ± 1,52 | | | 11 ± 1 | | 12,33 ± 1,15 | | - | |  |
| **C7** | 11,66 ± 1,52 | 12,66 ± 1,15 | 10,66 ± 0,57 | 10,66 ± 0,57 | | 9,33 ± 1,15 | | - | | 14,33 ± 1,52 | | 14,66 ± 0,57 | | 13,33 ± 0,57 | | | 12,33 ± 1,15 | | 13 ± 1 | | - | |  |
| **C11** | 10,66 ± 1,52 | 12,33 ± 1,52 | 11 ± 0 | 10 ± 0 | | 9 ± 0 | | - | | 13,66 ± 1,52 | | 14 ± 0 | | 14 ± 0 | | | 13,66 ± 1,15 | | 14,66 ± 1,52 | | - | |  |
| **C14** | 10,66 ± 0,57 | - | 9,66 ± 0,57 | 9 ± 0 | | 8 ± 1 | | - | | - | | - | | 9,66 ± 0,57 | | | 8,66 ± 0,57 | | - | | - | |  |
| **C17** | 13 ± 0 | - | 11,66 ± 1,52 | 12 ± 1 | | 10,66 ± 0,57 | | - | | - | | - | | 13,33 ± 0,57 | | | 14 ± 0 | | 11 ± 1 | | - | |  |
| **C18** | 14,33 ± 1,15 | 12,66 ± 1,15 | 12,33 ± 1,15 | 12,66 ± 1,15 | | 10,33 ± 1,15 | | - | | - | | - | | 17,33 ± 1,15 | | | 15,66 ± 0,57 | | 15,66 ± 0,57 | | - | |  |
| **C20** | 13,33 ± 1,15 | 14 ± 1 | 12,33 ± 0,57 | 12 ± 0 | | 10,33 ± 0,57 | | - | | - | | - | | 15,33 ± 0,57 | | | 13,33 ± 0,57 | | 17,66 ± 0,57 | | - | |  |
| **C21** | 14,33 ± 0,57 | 15,33 ± 0,57 | 13,66 ± 1,15 | 12,66 ± 1,52 | | 10 ± 0 | | - | | - | | - | | 16 ± 1,73 | | | 14 ± 1 | | 17,66 ± 1,52 | | - | |  |
| **C22** | 15,33 ± 0,57 | 16 ± 0 | 14,33 ± 1,52 | 13,66 ± 0,57 | | 11,33 ± 0,57 | | - | | - | | - | | 15,66 ± 0,57 | | | 13,66 ± 1,15 | | 17,33 ± 1,52 | | - | |  |
| **C23** | 14,66 ± 1,52 | 15,66 ± 1,15 | 14 ± 1 | 13 ± 0 | | 11,66 ± 0,57 | | - | | - | | - | | 17,66 ± 0,57 | | | 16 ± 1 | | 16,66 ± 1,15 | | - | |  |
| **C24** | 15 ± 0 | 16,66 ± 0,57 | 12,66 ± 1,15 | 12,33 ± 0,57 | | 11 ± 1 | | - | | - | | - | | 16 ± 0 | | | 15,66 ± 1,52 | | 16,66 ± 1,52 | | - | |  |
| **C25** | 15,66 ± 0,57 | 16,33 ± 0,57 | 14 ± 0 | 13,33 ± 0,57 | | 10,66 ± 1,15 | | - | | - | | - | | 16,66 ± 0,57 | | | 15 ± 1,73 | | 15,66 ± 1,52 | | - | |  |
| **C26** | 14,66 ± 0,57 | 15 ± 1 | 13,66 ± 0,57 | 13,66 ± 0,57 | | 10,66 ± 1,52 | | - | | - | | - | | 17,33 ± 2,08 | | | 15,66 ± 1,52 | | 17,33 ± 1,52 | | - | |  |
|  | **Gram Negative Bacteria** | | | | | | | | | | | | | | | **Gram Positive Bacteria** | | | | | | | |
|  | ***E. coli* ATCC25922** | ***E. coli* ATCC35218** | ***E. coli* KL.16** | | ***E. coli* KL16.2a** | | ***E. coli* MA isolate** | | ***E. coli* isolate** | | ***S. typhi*isolate** | | ***S. typhi*isolate** | | ***S aureus* ATCC29213** | | | ***S. epidermidis*** | | ***S. saprophyticus*** | | ***S. aureus* isolate** | |
| **M2** | 12 ± 2 | - | 10,66 ± 0,57 | | 11 ± 1 | | 9,33 ± 0,57 | | - | | 12 ± 1 | | 11,33 ± 0,57 | | 14,66 ± 1,15 | | | 14 ± 0 | | 17,66 ± 1,52 | | - | |
| **M21a** | 17,66 ± 1,52 | 12 ± 1 | 15,33 ± 0,57 | | 16,33 ± 1,52 | | 13,33 ± 1,52 | | - | | 12,66 ± 0,57 | | 11 ± 0 | | 22,66 ± 1,15 | | | 19,66 ± 2,08 | | 18,33 ± 2,08 | | - | |
| **M22** | 13,66 ± 1,52 | - | 12 ± 0 | | 12,66 ± 1,15 | | 10 ± 1,73 | | - | | - | | - | | 13,66 ± 1,15 | | | 11,66 ± 1,15 | | 14 ± 0 | | - | |
| **M27** | 12 ± 1 | - | 10 ± 0 | | 10,66 ± 1,15 | | 8,33 ± 0,57 | | - | | - | | - | | 12 ± 0 | | | 11,33 ± 1,52 | | - | | - | |
| **M28** | 11 ± 1,73 | - | 10,66 ± 0,57 | | 10,33 ± 0,57 | | 9 ± 0 | | - | | - | | - | | 11,66 ± 0,57 | | | 10,66 ± 2,08 | | - | | - | |
| **M32** | 8,33 ± 0,57 | 10,66 ± 1,15 | 8 ± 0 | | 8 ± 0 | | 7,33 ± 0,57 | | - | | 14 ± 1 | | 13,33 ± 0,57 | | 12 ± 1,73 | | | 14 ± 0 | | 12,33 ± 0,57 | | - | |
| **M77** | 14,33 ± 1,52 | 14 ± 1 | 14,66 ± 0,57 | | 15 ± 1 | | 12,66 ± 0,57 | | - | | 15,33 ± 1,15 | | 14,66 ± 0,57 | | 16 ± 2,64 | | | 19 ± 1 | | 14,66 ± 0,57 | | - | |
| **M79** | 14,66 ± 0,57 | 14 ± 1,73 | 15 ± 1 | | 14,66 ± 0,57 | | 12 ± 1 | | - | | 15,33 ± 0,57 | | 15,33 ± 1,15 | | 14 ± 0 | | | 17,33 ± 1,15 | | 14,66 ± 1,52 | | - | |
| **M98** | 11,66 ± 2,3 | - | 11,66 ± 0,57 | | 12,33 ± 0,57 | | 7 ± 0 | | - | | - | | - | | 12,33 ± 0,57 | | | 10,33 ± 0,57 | | - | | - | |
| **M101** | 13 ± 1 | - | 12,33 ± 0,57 | | 12,66 ± 0,57 | | 10,33 ± 0,57 | | - | | - | | - | | 10,33 ± 0,57 | | | 10 ± 0 | | - | | - | |
| **M117** | 14,66 ± 0,57 | - | 14 ± 1 | | 13,33 ± 1,52 | | 12 ± 0 | | - | | - | | - | | 11,33 ± 0,57 | | | 10,33 ± 1,15 | | - | | - | |
| **M124** | 16,33 ± 1,52 | 15,33 ± 1,15 | 15,66 ± 1,15 | | 16,66 ± 1,52 | | 14,66 ± 0,57 | | - | | 15,66 ± 0,57 | | 15,66 ± 1,15 | | 22,33 ± 0,57 | | | 18,33 ± 1,52 | | 17 ± 1 | | - | |

**Supplementary Table 2.** Characteristics of the bioactive bacterial isolates.

|  | **Morphology** | **Motility** | **Arrangement** | **Spores** | **Gram reaction** |
| --- | --- | --- | --- | --- | --- |
| B1, C5, C7, C11, M21a, M77, M79, M124 | Dry creamy irregular colonies with undulate edges | + | Pairs or chains | Terminal | + |
| C2 | Slimy white circular colonies with entire edges | - | Long chains | Terminal | + |
| C6 | Yellow to brown dry and adherent colonies with elevated ridges | + | Singles | - | - |
| C14 | Creamy large irregular colonies | + | Singles or pairs | Central | + |
| C17, C18, C21, C22, C23, C24, C25, C26 | Slightly yellowish, smooth and irregular colonies | + | Singles or pairs | Terminal | + |
| C20 | White with irregular margins | - | Long chains | - | + |
| M2, M27, M28, M98 | Mucoid creamy colonies with irregular borders | - | Very long chains | Central or terminal | + |
| M21, M22, M101, M117 | White irregular colonies with undulate edges | - | Pairs or chains | Terminal | + |
| M32 | White irregular colonies with a characteristic odor | - | Singles or pairs | - | + |

**Supplementary Table 3.**Composition of culture media tested for antibiotic production by *Bacillus* sp. M21a against *B. megaterium* and *E. coli*.

| **Culture medium** | **Medium composition (g/L)** | | **Reference** | ***B. megaterium^a^*** | ***E. coli^a^*** |
| --- | --- | --- | --- | --- | --- |
| **LB** | Peptone | 10 | Liu et al., 2015 | + | + |
|  | Yeast extract | 5 |  |  |  |
|  | NaCl | 10 |  |  |  |
| **M1** | Glucose | 2.5 | Liu et al., 2015 | + | - |
|  | Tryptone | 17 |  |  |  |
|  | Phytone | 3 |  |  |  |
|  | NaCl | 5 |  |  |  |
| **M2** | Glucose | 1 | Liu et al., 2015 | + | - |
|  | Peptone | 1.5 |  |  |  |
|  | Yeast extract | 3 |  |  |  |
|  | NaCl | 6 |  |  |  |
| **M3** | Tryptone | 6 | Liu et al., 2015 | + | - |
|  | Yeast extract | 3 |  |  |  |
|  | CaCl_2_ | 0.38 |  |  |  |
| **M4** | Glucose | 0.5 | Gulhane et al., 2014 | + | - |
|  | citric acid | 1 |  |  |  |
|  | MgSO_4_ | 0.2 |  |  |  |
|  | MnSO_4_ | 0.01 |  |  |  |
|  | KH_2_PO_4_ | 0.5 |  |  |  |
|  | K_2_HPO_4_ | 0.5 |  |  |  |
|  | FeSO_4_ | 0.01 |  |  |  |
| **M5** | Chitin | 0.1 | Gomashe et al., 2014 | + | - |
| **(Minimal medium)** | KH_2_PO_4_ | 0.01 |  |  |  |
|  | MgSO_4_.7H_2_O | 0.005 |  |  |  |
| **M6** |  |  |  |  |  |
|  | Peptone | 5 | Uddin et al., 2013 | + | - |
|  | Meat extract | 3 |  |  |  |
|  | NaCl | 5 |  |  |  |
| **M7** | Glucose | 0.1 | Uddin et al., 2013 | + | - |
|  | Yeast extract | 0.02 |  |  |  |
|  | NaCl | 0.05 |  |  |  |
| **M8** | Peptone | 0.025 | Uddin et al., 2013 | - | - |
|  | Meat extract | 0.075 |  |  |  |
|  | NaCl | 0.15 |  |  |  |
|  | KCl | 0.1 |  |  |  |
|  | MgCl_2_ | 0.1 |  |  |  |
|  | FeSO_4_ | 0.1 |  |  |  |
| **M9** | Glucose | 0.1 | Uddin et al., 2013 | + | - |
|  | NaCl | 0.05 |  |  |  |
| **M10** | Sucrose | 10 | Beric et al., 2012 | + | - |
|  | Peptone | 10 |  |  |  |
| **M11** | Glucose | 10 | Vijayalakshmi et al., 2011 | + | - |
|  | Triammonium citrate | 2.5 |  |  |  |
|  | MgSO_4_ | 0.2 |  |  |  |
|  | MnSO_4_ | 0.2 |  |  |  |
| **M12** | Glucose | 20 | Youcef Ali et al., 2004 | + | - |
|  | Peptone | 10 |  |  |  |
|  | Yeast extract | 10 |  |  |  |
| **M13** | Sucrose | 20 | Jacques et al., 1999 | + | - |
|  | Peptone | 30 |  |  |  |
|  | Yeast extract | 7 |  |  |  |
|  | KH_2_PO_4_ | 1.9 |  |  |  |
|  | Trace element solution | 1 mL/L |  |  |  |

^a^A (+) indicates if activity of the crude extract was observed against the test strain, (-): no activity.

**Supplementary Table 4.**^1^H and ^13^C NMR data, ^1^H–^1^H COSY, and HMBC correlations for the antibacterial compound of *Bacillus* sp. M21a.

| **Position** | **δ_H_ [ppm], *J*** | **mult.** | **δ_C_ [ppm]** | **^1^H - ^1^H COSY** | **^1^H - ^13^C HMBC** |
| --- | --- | --- | --- | --- | --- |
| 1 | 7.32 (ddd, *J* = 7.9, 7.1, 1.0 Hz, 2H) | CH | 129.78 | 2;6 | 2; 3; 5 |
| 2 | 7.61 (m, 2H) | CH | 130.162 | 1;3 | 4; 6 |
| 3 | 7.71 (m, 2H) | CH | 113.355 | 2 | 1; 5 |
| 4 |  | C | 143.329 |  | 2; 6 |
| 5 |  | C | 121.466 |  | 11 |
| 6 | 8.23 (dt, *J* = 7.9, 1.0 Hz, 1H) | CH | 122.528 | 1 | 2;4 |
| 7 |  | C | 132.264 |  | 11 |
| 8 |  | C | 130.738 |  | 6 |
| 9 |  | C | 137.133 | 11 | 10 |
| 10 | 8.47 (d, *J* = 4.9 Hz, 1H) | CH | 138.474 | 10 | 8;9 |
| 11 | 8.32 (d, *J* = 5.0 Hz, 1H) | CH | 120.12 |  | 5;7 |
| 12 |  | C | 203.078 |  |  |
| 13 | 2.83 (s) | CH_3_ | 25.946 |  | 9 |

**References**

Berić, T., Kojić, M., Stanković, S., Topisirović, L., Degrassi, G., Myers, M., ...and Fira, D. (2012). Antimicrobial activity of *Bacillus* sp. natural isolates and their potential use in the biocontrol of phytopathogenic bacteria. *Food Technology and Biotechnology*, *50*(1), 25-31.

Gomashe, A. V., Sharma, A. A., and Wankhede, M. A. (2014). Screening and Evaluation of Antibacterial Activity of Bacteriocin Producing LAB against some Selected Bacteria Causing Food Spoilage. *Int. J. Curr. Microbiol. App. Sci*, *3*(8), 658-665.

Gulhane, P. A., Gomashe, A. V., and Lade, S. (2014). Optimization of Bacitracin Production from *Bacillus licheniformis* NCIM 2536. *Int. J. Curr. Microbiol. App. Sci*, *3*(9), 819-829.

Jacques, P., Hbid, C., Destain, J., Razafindralambo, H., Paquot, M., De Pauw, E., &Thonart, P. (1999). Optimization of biosurfactant lipopeptide production from *Bacillus subtilis* S499 by Plackett-Burman design. In *Twentieth Symposium on Biotechnology for Fuels and Chemicals* (pp. 223-233). Humana Press, Totowa, NJ.

Liu, C., Sheng, J., Chen, L., Zheng, Y., Lee, D. Y. W., Yang, Y., ...and Shen, L. (2015). Biocontrol activity of *Bacillus subtilis* isolated from *Agaricusbisporus* mushroom compost against pathogenic fungi. *Journal of agricultural and food chemistry*, *63*(26), 6009-6018.

Uddin, M., Mahmud, N., Anwar, N. and Manchur, M. A. (2013). Bioactive metabolite production by *Streptomyces albolongus* in favourable environment. *Journal of Microbiology and Infectious Diseases*, *3*(02).

Vijayalakshmi, S., Kumar, V. and Thankamani, V. (2013). Optimization and cultural characterization of *Bacillus* RV. B2. 90 producing alkalophilic thermophilic protease. *Research Journal of Biotechnology*, *8*(5), 37-43.

Youcef-Ali, M., Chaouche, N. K., Dehimat, L., Bataiche, I., Mounira, K. A. R. A., Cawoy, H. E., and Thonart, P. (2014). Antifungal activity and bioactive compounds produced by *Bacillus mojavensis* and *Bacillus subtilis*. *African Journal of Microbiology Research*, *8*(6), 476-484.
